# DEB-TACE combined with hepatic artery infusion chemotherapy might be an affordable treatment option for advanced stage of HCC

**DOI:** 10.1038/s41598-022-21472-1

**Published:** 2022-10-07

**Authors:** Yasuteru Kondo, Tatsuki Morosawa, Soichiro Minami, Yasuhito Tanaka

**Affiliations:** 1 Department of Hepatology, Sendai Kousei Hospital, Sendai, Japan; 2 Department of Gastroenterology, Kumamoto University Hospital, Kumamoto, Japan

**Keywords:** Cancer therapy, Cancer, Liver cancer

## Abstract

Alternative treatment modalities are necessary because of the low response rates and unsuitability of molecular-targeted agents (MTA) and/or immune checkpoint inhibitors (iCIs) in HCC patients. Therefore, we analyzed whether drug-eluting beads (DEB)-transcatheter arterial chemoembolization (TACE) with low-dose-FP (Ultra-FP) therapy could improve the efficacy and safety of treatment in difficult-to-treat HCC patients, especially those with advanced stage HCC. From November 2017 to April 2021, 118 consecutive patients with non-resectable difficult-to-treat HCC were included in this study. All patients were treated with Ultra-FP therapy. After the weak DEB-TACE procedure, we administered low-dose FP for 2 weeks followed by resting for 4 weeks. The numbers of HCC patients CR/PR/SD/PD induced by Ultra-FP therapy were 36/52/17/13 (Modified RECIST) patients, respectively. The objective response rate of Ultra-FP therapy was 74.6% (88/118 patients). Tumor marker reduction was observed in 81.4% (96/118 patients). The objective response rate (ORR) in the HCC patients with portal vein tumor thrombosis (PVTT) was 75% (18/24 patients). Median overall survival (mOS) of all included HCC patients was 738 days. The mOS of HCC patients with PVTT (−)/PVTT (+) was 816 days/718 days. The proportion of patients based on ALBI grade system was not significantly different between pre- and after 3 course Ultra-FP therapy. Ultra-FP therapy might be an affordable treatment option for difficult-to-treat advanced HCC. ORR and overall survival after receiving Ultra-FP therapy were remarkable in comparison to various kinds of systemic therapy including MTA and iCIs.

## Introduction

Hepatocellular carcinoma (HCC) is the sixth most common malignant cancer and the second leading cause of cancer deaths worldwide^1^. The treatment efficacy for patients with advanced-stage HCC has been improved by molecular-targeted agents (MTA), immune check point inhibitors (iCIs), and radiation therapy, in addition to TACE and/or hepatic arterial infusion chemotherapy (HAIC)^2–6^. However, the treatment efficacy for patients with advanced-stage HCC treated by a single agent has not been adequate^2–4^. iCIs and/or MTA including sorafenib, lenvatinib, regorafenib and cabozantinib etc. are standard treatments according to current international guidelines^7^. However, alternative treatment modalities are required because of the low response rates and unsuitability of MTA and iCIs in the real world.

Combinations and/or sequential treatments with various agents have been carried out to improve the treatment efficacy for patients with advanced-stage HCC^8,9^. It was reported that the treatment efficacy of TACE for patients with up-to-seven-out in the intermediate and advanced stage HCC was not adequate^10^. It has been reported that TACE with drug-eluting beads (DEB-TACE) showed a higher complete response rate, objective response rate and overall survival time with fewer common adverse events than conventional TACE (cTACE) in some groups^11,12^. However, the other group reported that the DEB-TACE and the cTACE are equally effective and safe, with the advantage of DEB-TACE causing less post-procedural abdominal pain^13^.

The pharmacokinetics of HAIC were based on the theories of “first pass effect” and “increased local concentration”^14–16^. In HAIC, a highly concentrated chemotherapeutic drug is injected into the liver tumor and surrounding area via the hepatic artery. A high concentration of a chemotherapeutic drug in the tumor site could induce an efficient anti-tumor effect^17^. Moreover, fewer systemic side effects occurred due to the “first pass effect” of the liver^18^. Recently, many groups, especially in Asia, have reported the effectiveness of treatments with HAIC in patients with advanced stage HCC^19–23^. In comparison to sorafenib therapy, HAIC might have a superior effect for advanced stage HCC, especially, with a portal vein tumor thrombus (PVTT)^24^. Other groups reported that intrahepatic tumor reduction by HAIC significantly prolonged the survival of patients, irrespective of PVTT or initial distant metastasis^25^.

In addition to the conventional methods of HAIC, novel methods of HAIC have been proposed by many groups^19,23,26–28^. Dr. Nagamatsu et al. reported that 5-fluorouracil (5-FU) HAIC with cisplatin suspension in lipiodol (New-FP) could be effective for HCC patients with PVTT^27^. Moreover, it has been reported that New-FP could prolong overall survival (OS) compared to sorafenib by using propensity score matching^26^. Dr. Guo et al. reported the efficacy and safety of TACE followed by HAIC for treating advanced HCC^6^.

We modified the treatment regimens to improve the efficacy of DEB-TACE and HAIC for intermediate and advanced HCC as described above. In this study, we analyzed whether Ultra-FP therapy (combination weak embolization with DEB-TACE (CDDP) and HAIC (low dose FP: CDDP and 5-FU)) could improve the efficacy and safety of treatment in difficult-to-treat HCC patients, especially those with advanced stage HCC.

## Methods

### Study design and inclusion criteria

**Figure 1:**
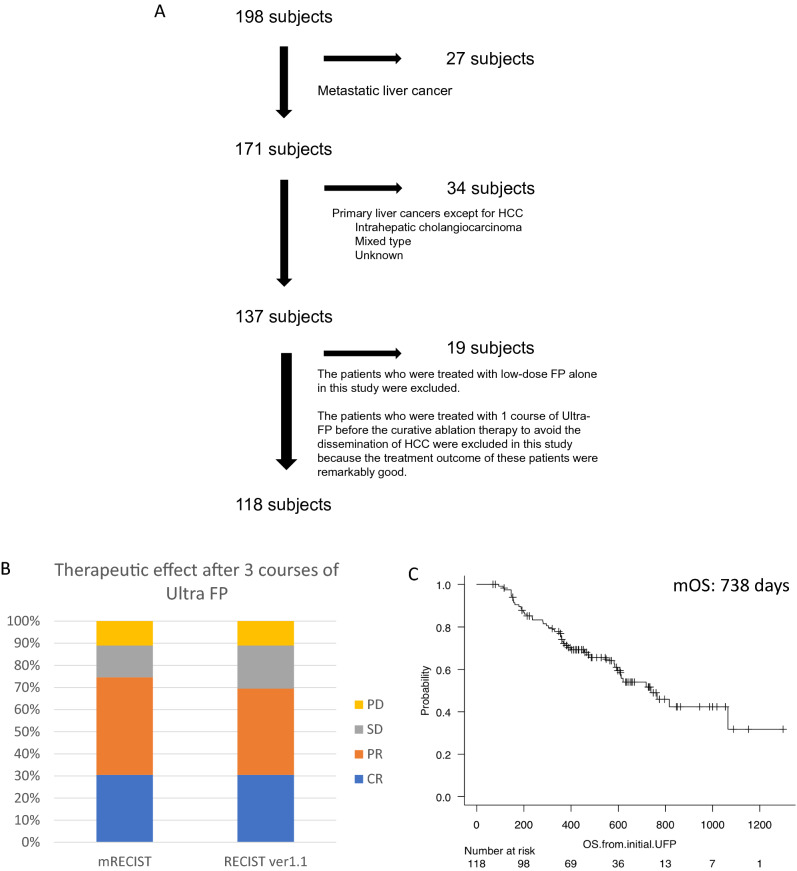
Flow chart of patients included in this study and survival and treatment response of Ultra FP therapy. Flow chart of patients included in this study is shown (**A**). Treatment response of all included patients treated by Ultra-FP therapy (modified Response Evaluation Criteria in Solid Tumors (mRECIST) and RECIST ver1.1) is shown (**B**). The survival curve of included patients analyzed by Kaplan–Meier method is shown (**C**). mOS of all included patients (118 patients) was 738 days (24 months) (**C**).

This study was approved by the Ethics Committee of Sendai Kousei Hospital in accordance with the Declaration of Helsinki and written informed consent was obtained from all subjects. This study was a single center and retrospective observational study. The protocol of chemotherapy was also approved by the Ethics Committee of Sendai Kousei Hospital. From November 2017 to May 2021, 198 patients had been treated with HAIC. Eighty patients with metastatic liver cancer, performance status 3–4, BCLC D stage, low-dose-FP without DEB-TACE, and other liver cancers such as cholangiocarcinoma were excluded in this study (Fig. 1A). One hundred eighteen consecutive patients with non-resectable, difficult-to treat HCC (multiple TACE-refractory HCC, Treatment responses of MTAs were PD, Huge HCC with multiple intra/extra hepatic metastasis, multiple HCC beyond the up-to-seven criteria, HCC with macroscopic vascular invasion) were included in this study (Fig. 1A, Table 1). The following inclusion criteria were used: (1) HCC diagnosed by tumor biopsy or radiological evaluation using dynamic enhanced computed tomography (CT) and/or gadolinium-ethoxybenzyl-diethylenetriamine pentaacetic acid (EOB) enhanced-magnetic resonance imaging (MRI) combined with tumor markers: alpha-fetoprotein (AFP) and des-γ-carboxy prothrombin (DCP); (2) age > 20 years, and (3) patients treated with Ultra-FP as a multidisciplinary treatment in progressed HCC.

**Table 1:** Baseline characteristics.

Age (years)*	72 (9)
Sex (male/female)	94/24
Etiology (HBV/HCV/HBV + HCV/HBV + Alcohol/Alcohol/NASH/Alcohol + Mets/others)	12/30/1/4/32/13/3/23
Child–Pugh classification (A/B)	93/25
Child–Pugh score (5/6/7/8/9)	60/33/11/8/6
ALBI grade (1/2a/2b/3)	34/28/44/12
ALBI score*	− 2.23 (0.61)
Albumin (g/dl)*	3.6 (0.7)
Total bilirubin (mg/dl)*	1.06 (0.62)
Prothrombin time (%)*	93 (16)
HCC staging (at the first Ultra FP) Stage# (II/III/IVA/IVB)	25/45/26/22
UICC Stage (IB/II/IIIA/IIIB/IVA/IVB)	14/41/12/20/9/22
BCLC Stage (A/B/C)	15/47/56
BCLC Stage with Kinki criteria (A/B1/B2/B3/C)	15/16/25/6/56
MVI (Vp 2/3/4, Vv 2/3)	10/7/7, 5/4
Treatment before ultra FP (operation/TACE and/or Ablation/TKI/none)	13/65/12/49
TKI before ultra FP (sorafenib/lenvatinib)	6/9

*UICC* Union for International Cancer Control staging, *BCLC* Barcelona clinic liver cancer, *MVI* macroscopic vascular invasion, *TKI* tyrosine kinase inhibitor.

*Mean(SD) #general rules for the clinical and pathological study of primary liver cancer staging.

Clinical data including age, sex, etiology of HCC, Child–Pugh score, albumin-bilirubin (ALBI) score, treatment history of HCC, treatment history of partial splenic embolization (PSE) and the presence of macroscopic vascular invasion (MVI) and extrahepatic spread (EHS). HCC was classified using the General Rules for the Clinical and Pathological Study of Primary Liver Cancer staging, Union for International Cancer Control (UICC) staging, Barcelona Clinic Liver Cancer (BCLC) staging and BCLC staging with Kinki criteria^10,29^.

### Data evaluation

The following evaluation items were analyzed: (1) Overall Survival (OS) after receiving Ultra-FP treatment. (2) Tumor response rate after receiving 3 courses of Ultra-FP treatment using modified (Response Evaluation Criteria in Solid Tumors) RECIST criteria and RECIST version 1.1. (3) Adverse events induced by Ultra-FP using Common Terminology Criteria for Adverse Events (CTCAE) Ver 5.0. If the patients had been treated with iCIs after Ultra-FP, they were censored to avoid the effects of iCIs.

### Treatment protocol of Ultra-FP

All patients were treated by weak embolization (maximum 30% embolization of HCC) using Hepasphere with CDDP (Kondo 3 catheter and Michibiki, Hanaco Medical, Tokyo Japan). We carried out angiography to evaluate the region of embolization during the DEB-TACE. After the weak DEB-TACE procedure, we immediately carried out catheter implantation. Some patients needed gastroduodenal artery coiling and/or right gastric artery coiling using metallic coils to avoid gastroduodenal ulcer or pancreatitis. A five-French catheter (Anthron P-U Catheter; Toray Medical Co. Ltd., Tokyo, Japan) was inserted in the proper hepatic artery or targeted for a more specific hepatic artery. On day 1 we carried out weak DEB-TACE with CDDP and low dose FP (injection of 250 mg of 5FU and 2–8 mg of CDDP using injection pump). Low dose FP was carried out 10 times for 2 weeks followed by a rest for 4 weeks. This regimen is the course of Ultra-FP. This regimen was continued until the appearance of severe AE, tumor progression, or a remarkable effect of treatment and conversion to curative therapy (Liver resection or RFA/MWA ablation).

### Statistical analysis

All statistical analyses were carried out using JMP statistical analysis software (JMP Pro version 15, SAS Institute Inc., Cary, NC, USA). The survival time was calculated using Kaplan–Meier method and the analysis of log-rank test.

## Results

### Baseline characteristics of patients treated with Ultra-FP therapy

One hundred eighteen patients were involved in this study (Fig. 1A and Table1). The mean age was 72 years. Most of the HCC etiology was non-HBV and/or non-HCV-related HCC (71 patients/118 patients, respectively). A treatment history for HCC existed in 71 patients including resection, TACE, RFA/MWA ablation and MTA (Table 1). The numbers of patients based on the stage of the general rules for the clinical and pathological study of primary liver cancer, I/II/III/IVA/IVB, were 0/25/45/26/22 patients, respectively. All patients of stage II were TACE refractory patients.

The numbers of patients based on the HCC BCLC staging A, B, C and D system were 15/47/56/0 patients, respectively. Fifteen cases of BCLC A stage patients were treated with Ultra-FP. Three out of 15 patients had recurrence of HCC after liver resection. Then, these three patients were treated with TACE combined with RFA. However, these patients had TACE-refractory HCC. Ten out of 15 patients were TACE-refractory or TACE combined with RFA-refractory patients. Two out of 15 patients had poorly differentiated HCC and infiltrative tumor. Sixteen patients of BCLC B1 patients were treated with Ultra-FP. Ten of 16 patients were TACE-refractory patients after several TACE sessions. Three out of 16 patients had TACE combined with Lenvatinib-refractory HCCs after receiving liver resection. Three out of 16 patients had poorly differentiated HCC and infiltrative tumors. The presence of portal vein tumor thrombosis (PVTT) was detected in 24 of 118 patients. The level of the liver reserve was evaluated by the Child–Pugh classification and ALBI grade system. The numbers of patients based on the Child–Pugh classification, A/B/C, were 93/25/0 patients, respectively. The numbers of patients based on the ALBI grade system, 1/2a/2b/3 were 34/28/44/12 patients, respectively (Table 1).

### Treatment effect of Ultra-FP therapy and adverse events

The treatment effect of Ultra-FP therapy was evaluated by the modified RECIST and RECIST ver1.1 after 3 courses of therapy (Table 2 and Fig. 1B). The numbers of HCC patients, CR/PR/SD/PD (modified RECIST), induced by Ultra-FP therapy were 36/52/17/13 patients, respectively. The objective response rate (ORR) of Ultra-FP therapy was 74.6% (88/118 patients) and the disease control rate (DCR) was 89.0% (105/118 patients). Tumor marker reduction (AFP or DCP) was observed in 81.4% (96/118 patients). ORR in the patients with PVTT was 75% (18/24 patients). ORR in the patients with an extrahepatic lesion was 64.7% (11/17 patients). Median overall survival (mOS) of all included HCC patients was 738 days (Fig. 1C). Nine out of 13 PD patients received post-treatments. Four out of 9 patients were treated with Lenvatinib. Three out of 9 patients were treated with TACE, RFA and radiation. Two out of 9 patients were treated with radiation. Among the patients that achieved CR or PR, one patient was treated with liver resection and the others were treated with RFA or MWA after reducing the size of HCCs (< 5 cm). We considered continuing the courses of Ultra-FP depending on the response, adverse events and tolerability after 3 courses of Ultra-FP. Sixty-eight patients received 3–5 courses of Ultra-FP. Twenty-one patients received 6–8 courses of Ultra-FP. Eighteen patients received 8–10 courses of Ultra-FP. Eleven patients received more than 11 courses of Ultra-FP. Some patients received several courses of Ultra-FP for the recurrence of HCC after achieving CR by Ultra-FP.

**Table 2:** Evaluation of treatment response after 3 courses of Ultra FP therapy from initial Ultra FP therapy.

	RECIST ver1.1	Modified RECIST
Treatment effect (CR/PR/SD/PD)	36/46/23/13	36/52/17/13
Response rate (CR + PR)	69.5% (82/118)	74.6% (88/118)
Tumor control probability (CR + PR + SD)	89.0% (105/118)	89.0% (105/118)
Median overall survival time (days)	738	
Reduction of tumor marker	81.4% (96/118)	
Response rate in the patients with portal vein tumor thrombosis	66.7% (16/24)	75% (18/24)
Response rate in the patients with extrahepatic lesions	58.8% (10/17)	64.7% (11/17)

Grade 1/2 adverse events after initial Ultra-FP therapy were analyzed using CTCAE ver 5.0. Some patients experienced abdominal pain, fever, malaise, nausea and/or vomiting, anorexia, diarrhea, hypertension, increased creatinine, anemia, decreased platelet count (Table 3). Grade 3/4 adverse events induced by tumor lysis (asparate aminotransferase increased, alanine aminotransferase increased, blood bilirubin increased, GGT increased and hypoalbuminemia) were temporarily observed in some patients (Table 3). However, there were no severe adverse events that might have resulted in death.

**Table 3:** Adverse events of UFP therapy.

n (%)	Any grade	Grade 2	Grade 3/4
Abdominal pain	18 (15)	5 (4)	0
Fever	55 (47)	7 (6)	0
Malaise	6 (5)	0	0
Nausea and/or Vomiting	8 (7)	0	0
Anorexia	17 (14)	6 (5)	0
Diarrhea	6 (5)	5 (4)	0
Hypertension	88 (75)	23 (19)	0
Aspartate aminotransferase increased	99 (84)	0	34 (29)
Alanine aminotransferase increased	63 (54)	11 (9)	11 (9)
Blood bilirubin increased	58 (49)	21 (18)	6 (5)
GGT increased	78 (66)	24 (20)	12 (10)
Hypoalbuminemia	92 (78)	29 (25)	13 (11)
Creatinine increased	54 (46)	0	0
Anemia	75 (64)	6 (5)	0
Platelet count decreased	42 (36)	18 (15)	0

CTCAE ver.5.0.

### Subgroup analysis of mOS due to the tumor condition and liver functional reserve

**Figure 2:**
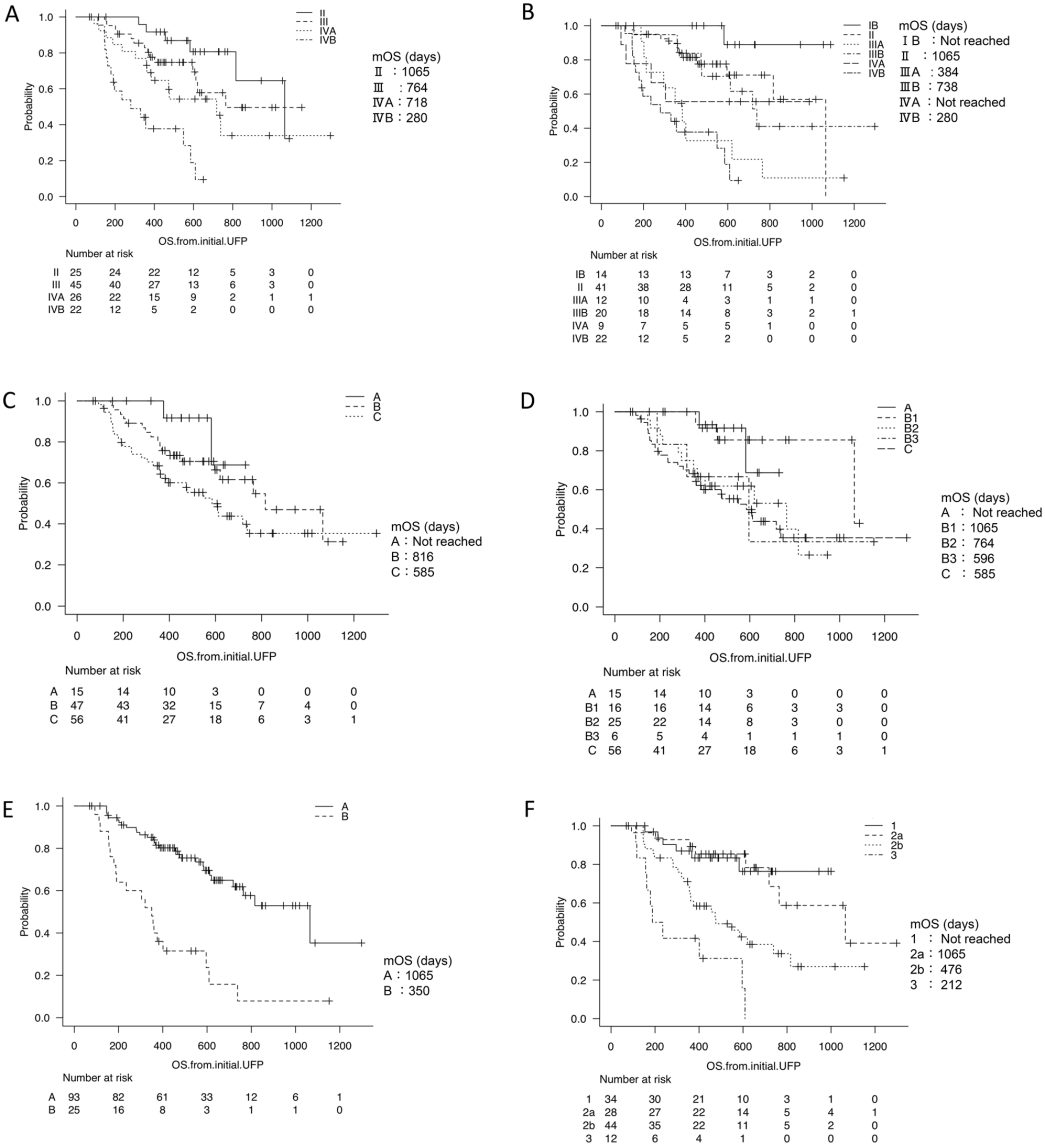
Survival time depending on the various kinds of tumor staging systems and the liver functional reserve. The survival curves of the tumor staging systems based on the general rules for the clinical and pathological study of primary liver cancer (**A**), Union for International Cancer Control (UICC) (**B**), Barcelona Clinic Liver Cancer (BCLC) (**C**) and Barcelona Clinic Liver Cancer (BCLC) with Kinki criteria (**D**) are shown. mOS stage based on the general rules for the clinical and pathological study of primary liver cancer, I/II/III/IVA/IVB, was NA/1065/764/718/280 days, respectively (**A**). mOS of UICC staging, IB/II/IIIA/IIIB/IVA/IVB, was Not reached/1065/384/738/Not reached/280 days, respectively (**B**). mOS of BCLC stage, A/B/C, was Not reached/816/585 days, respectively (**C**). mOS of a sub-classification of BCLC (Kinki criteria) stage, A/B1/B2/B3/C, was Not reached/1065/764/596/585 days, respectively (**D**). The survival curves of liver functional reserve based on Child–Pugh (**E**) and ALBI grade (**F**) systems are shown. mOS of HCC patients with Child–Pugh A/B/C was 1065/350 days, respectively (**E**). Moreover, the mOS of HCC patients with albumin-bilirubin (ALBI) grade 1/2a/2b/3 was Not reached/1065/476/212 days (**F**).

At first, we analyzed the subgroup of HCC patients depending on the various kinds of tumor staging systems. The mOS stage based on the general rules for the clinical and pathological study of primary liver cancer, I/II/III/IVA/IVB, was NA/1065/764/718/280 days, respectively (Fig. 2A). The mOS of UICC staging, IB/II/IIIA/IIIB/IVA/IVB was Not reached/1065/384/738/NA/280 days, respectively (Fig. 2B). The mOS of BCLC stage A/B/C was Not reached/816/585 days, respectively (Fig. 2C). The mOS of the sub-classification of BCLC (Kinki criteria) stage A/B1/B2/B3/C was Not reached/1065/764/596/585 days, respectively (Fig. 2D).

Then, we analyzed the subgroup of HCC patients depending on the criteria of the liver functional reserve. The mOS of the HCC patients in the Child–Pugh A/B/C were 1065/350/NA days, respectively (Fig. 2E). Moreover, the mOS of the HCC patients with ALBI grade 1/2a/2b/3 were NA/1065/476/212 days, respectively (Fig. 2F).

**Figure 3:**
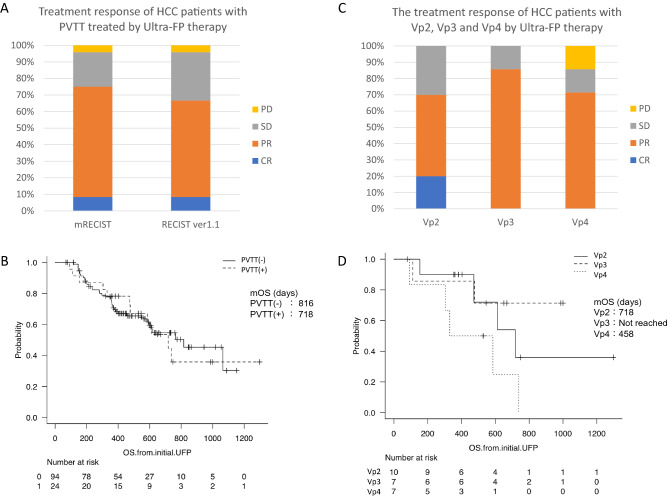
Treatment response and survival time depending on the existence of PVTT. Treatment response of HCC patients with portal vein tumor thrombosis (PVTT) treated by Ultra-FP therapy (modified Response Evaluation Criteria in Solid Tumors (RECIST) and RECIST ver 1.1) is shown (**A**). The survival curves of HCC patients with or without PVTT are shown (**B**). The treatment response of HCC patients with PVTT by Ultra-FP therapy CR/PR/SD/PD was 2/16/5/1 patients, respectively (**A**). The mOS of HCC patients with PVTT (−)/PVTT (+) was 816 days/718 days, respectively (**B**). The treatment response of HCC patients with portal vein invasion grade Vp2, Vp3 and Vp4 based on general rules for the clinical and pathological study of primary liver cancer by Ultra-FP therapy are shown (**C**). Vp4, main portal vein invasion; Vp3, first branch portal vein invasion; Vp2, second branch portal vein invasion. The treatment response of HCC patients with Vp2 by Ultra-FP therapy CR/PR/SD/PD was 2/5/3/0 patients, respectively (**C**). The treatment response of HCC patients with Vp3 by Ultra-FP therapy CR/PR/SD/PD was 0/6/1/0 patients, respectively (**C**). The treatment response of HCC patients with Vp4 by Ultra-FP therapy CR/PR/SD/PD was 0/5/1/1 patients, respectively (**C**). The survival curves of HCC patients with Vp2/Vp3/Vp4 are shown (**D**). The mOS of HCC patients with Vp2/Vp3/Vp4 was 718/Not reached/458 days, respectively (**D**).

Finally, we analyze the subgroup of HCC patients depending on the existence of PVTT. The treatment response of HCC patients with PVTT by Ultra-FP therapy, CR/PR/SD/PD (modified RECIST), were 2/16/5/1 patients, respectively (Fig. 3A). The response rate of HCC patients with PVTT was 75%. The survival curves of HCC patients with or without PVTT were almost the same (Fig. 3B). The mOS of HCC patients with PVTT (−)/PVTT (+) was 816 days/718 days. The treatment responses of the HCC patients with Vp2, Vp3 and Vp4 by Ultra-FP therapy are shown in Fig. 3C. The treatment responses of HCC patients with Vp4 PVTT by Ultra-FP therapy, CR/PR/SD/PD (modified RECIST), are 0/5/1/1 patients, respectively (Fig. 3C). The mOS of HCC patients with Vp2/Vp3/Vp4 were 718/Not reached/458 days, respectively (Fig. 3D).

### Analysis of liver functional reserve during Ultra-FP therapy

**Figure 4:**
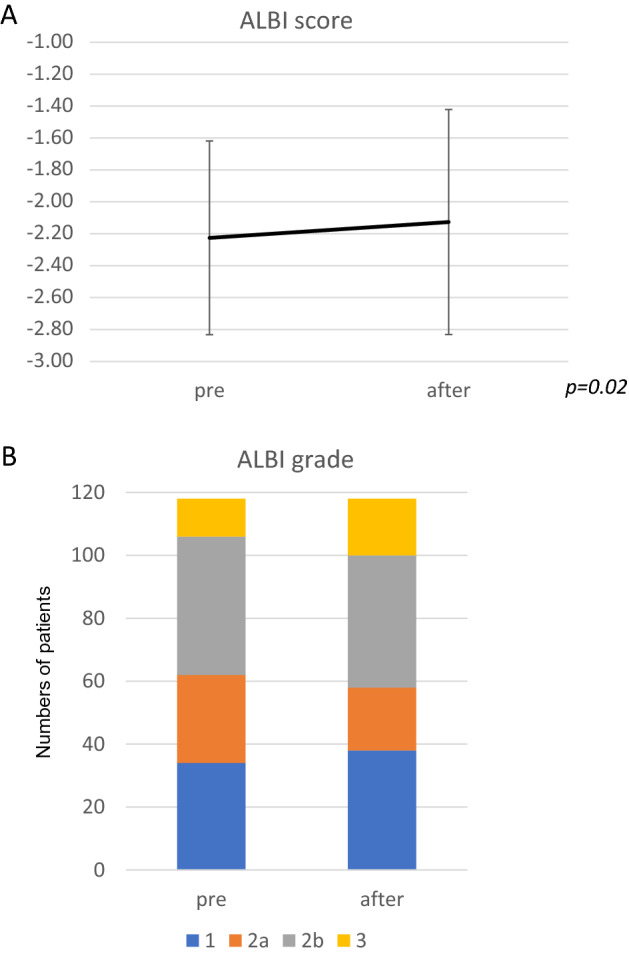
Analysis of liver functional reserve during Ultra-FP therapy. Albumin-bilirubin (ALBI) scores before and after 3 course of Ultra-FP therapy are shown (**A**). Y-axis indicates ALBI score. Error bars indicate standard deviation. The numbers of patients based on ALBI grade system at the pre-treatment and after 3 course of Ultra-FP therapy are shown (**B**).

A comparison of ALBI scores between before and after 3 courses of Ultra-FP therapy was carried out (Fig. 4A). ALBI scores were significantly increased during 3 courses of Ultra-FP therapy (*p* = *0.02*). However, the change of the mean score was 0.1 (pre = − 2.23: after = − 2.13) (Fig. 4A). The numbers of patients based on the ALBI grade system at pre-treatment, 1/2a/2b/3 were 34/28/44/12 patients, respectively (Fig. 4B). The numbers of patients based on the ALBI grade system after 3 courses of Ultra-FP therapy, 1/2a/2b/3 were 38/20/42/18 patients, respectively. The proportion of patients based on the ALBI grade system was not significantly different between pre and after 3 courses of Ultra-FP therapy (Fig. 4B).

## Discussion

The treatment efficacy of the patients with advanced-stage HCC and difficult-to-treat intermediate stage HCC has been improved by MTA, iCIs, and radiation therapy in addition to TACE and/or HAIC. However, the treatment efficacy by a single agent of has not been adequate. Some groups, including ours, developed modified methods of HAIC that contributed to a better treatment response, less liver damage and fewer adverse events. In this study, we developed Ultra-FP therapy that combined weak embolization by DEB-TACE with low-dose FP HAIC. The HCC patients involved in this study had remarkably severe tumors and liver reserve in comparison to the SHARP trial, REFLECT trial and IMbrave150 trial, since we included Child–Pugh B patients and patients with a treatment history of MTA and/or TACE/RFA/liver resection^2–4^. Therefore, when we analyzed HCC patients with Child–Pugh A liver reserve, the mOS was 1065 days in the patients treated by Ultra-FP therapy. Moreover, the mOS of Child–Pugh B patients was 350 days. These results showed that the mOS of Child B HCC patients treated with Ultra-FP therapy had a similar mOS to Child–Pugh A HCC patients treated with MTA^3,4^. Moreover, we analyzed the survival of HCC by using BCLC staging with the Kinki criteria, which might well differentiate the conditions among BCLC B^10,29^. In our data, the survival of HCC patients treated with Ultra-FP therapy differed among BCLC B1, B2 and B3. BCLC-B2 stage might include a wide variety of HCC. Nine cases out of 25 patients were solely DEB-TACE refractory patients. Three out of 25 patients were DEB-TACE refractory patients after receiving liver resection at the initial treatment. We included TACE refractory patients since we thought that DEB-TACE combined with HAIC was able to decrease the tumor volume in TACE-refractory patients since weak DEB-TACE might induce partial tumor necrosis by blocking the tumor blood supply. A partial necrotic tumor might induce tumor immunity and enhance the effect of HAIC. However, DEB-TACE before HAIC might block the infused drug get into the tumor terminal vessels, and in some extent decrease the therapeutic effect of HAIC. We should evaluate the effect of HAIC without DEB-TACE among the TACE-refractory patients in the future study. Twelve cases of 25 patients were treatment-naïve and had giant HCC and/or multiple HCCs. One case out of 25 patients had poor functional liver reserve after repeated TACE. We thought these patients were difficult-to-treat by DEB-TACE alone. We could not choose DEB-TACE alone among the same conditions of HCC in the Ultra-FP treatment group of BCLC-B2 since most of the 25 patients were inappropriate for DEB-TACE alone. Therefore, we could not compare the results between Ultra-FP group and the solely DEB-TACE group. The survival of BCLC B3 HCC patients treated with Ultra-FP therapy showed a better prognosis compared to previous reports^10^. Therefore, Ultra-FP therapy was effective for treating advanced stage HCC in patients with poor liver reserve.

Previous reports indicated that HCC patients with PVTT treated with sorafenib had a poor prognosis^30^. However, the treatment response of HCC patients with PVTT by Ultra-FP therapy was remarkably high. The ability of Ultra-FP therapy to reduce the tumor volume might maintain the liver reserve since a reduction of PVTT could improve the blood supply of the background liver. Dr. Zheng et al. reported that sorafenib plus HAIC (oxaliplatin followed by 5-fluorouracil) had a better OS than sorafenib therapy in HCC with PVTT patients^31^. It has been reported that mOS was 16.3 months with sorafenib plus HAIC in HCC with Vp3/4. Moreover, Dr. He et al. reported a randomized clinical trial for the comparison between sorafenib plus HAIC (FOLFOX) and sorafenib therapy in HCC with PVTT patients^32^. It has been reported that the mOS was 13.7 months in sorafenib plus HAIC group versus 7.13 months in the sorafenib group. In our study, the mOS was 24.6 months with Ultra-FP in HCC with VP3/4 (14 patients). However, the sample size was relatively small in our study. We should analyze whether sorafenib plus Ultra-FP might improve the mOS in a future study. Some groups indicated that radiation therapy with HAIC could improve the treatment response of HAIC with PVTT^33,34^. These results suggested that Ultra-FP therapy with radiation therapy might be a better option for HCC with PVTT than Ultra-FP therapy alone.

It has been reported that atezolizumab and bevacizumab combination therapy had a better treatment response and OS than sorafenib therapy. However, various kinds of irAEs and poor liver reserve might restrict the usage of atezolizumab and bevacizumab combination therapy. The iCIs including atezolizumab might maintain the function for the re-activation of tumor immunity for several months. Therefore, Ultra-FP therapy as the post-treatment after atezolizumab and bevacizumab combination therapy could be a candidate treatment to enhance the tumor immunity since the cytotoxic effect of Ultra-FP therapy is quite strong. Immunogenic cell death induced by ferroptosis, necroptosis, and pyroptosis could enhance the anti-tumor immunity in patients treated by iCIs^35,36^. In addition to the strong cytotoxic effect of Ultra-FP therapy, the Ultra-FP therapy could maintain the liver reserve in this study. The atezolizumab and bevacizumab regimen became available in November 2020. If the patients had been treated with iCIs after Ultra-FP, they were censored to avoid the effects of iCIs in this study. Nine out of 56 patients in BCLC-C stage were treated with Ultra-FP after PD therapy evaluation of tyrosine kinase inhibitors (TKIs). Twenty-nine out of 56 patients were treated with TKIs (23 patients received Lenvatinib, 14 patients received Sorafenib, 2 patients received Ramucirumab) for several months after the induction of Ultra-FP therapy. However, the duration and/or durability of TKIs treatment after Ultra-FP therapy were limited due to adverse events in all included patients. The combination of TKIs and iCIs with Ultra-FP therapy for HCC patients with good liver reserve should be analyzed in the future study. Some studies have shown that the combination of MTA and iCIs could be more effective than monotherapy^37^. However, the combination of MTA and iCIs therapy requires adequate liver reserve.

Technical training for Ultra-FP therapy is necessary for doctors who lack experience in catheter therapy. The limitation of technical expertise in Ultra-FP therapy for liver cancer might be overcome in high volume treatment centers. This study was a single center and retrospective observational study. Therefore, we need to analyze whether Ultra-FP therapy could be superior to DEB-TACE or HAIC alone in multiple centers.

Some groups reported that various kinds of biomarkers could predict the prognosis for advanced HCC treated with HAIC^38–45^. Previously, we reported that myeloid derived suppressor cells (MDSCs) might contribute to the immunopathogenesis of HCC and affect the recurrence of HCC^46^. Dr. Mizukoshi et al. reported that the frequency of MDSCs before treatment was a prognostic factor in HAIC against HCC^38^. Immunological analysis of biomarkers is important since multidisciplinary treatments including iCIs, MTAs, radiation and Ultra-FP therapy etc. might improve the prognosis of HCC patients. Biomarkers for the treatment response of Ultra-FP therapy should be analyzed in the near future.

In conclusion, Ultra-FP therapy could be an affordable treatment option for difficult-to-treat advance HCC. ORR and OS after receiving Ultra-FP therapy were remarkable in comparison to various kinds of systemic therapy including MTA and iCIs. Maintaining the liver reserve might contribute to enabling various kinds of treatment. We need to determine the best combination therapy with Ultra-FP since many systemic therapies after or during Ultra-FP therapy might contribute to the stabilization of HCC.

## Data Availability

The datasets used and analyzed during the current study are available from the corresponding author on reasonable request.

## References

[1] Shiratori Y, Yoshida H, Omata M (2001). Management of hepatocellular carcinoma: Advances in diagnosis, treatment and prevention. Expert Rev. Anticancer Ther..

[2] Finn RS (2020). Atezolizumab plus bevacizumab in unresectable hepatocellular carcinoma. N. Engl. J. Med..

[3] Kudo M (2018). Lenvatinib versus sorafenib in first-line treatment of patients with unresectable hepatocellular carcinoma: A randomised phase 3 non-inferiority trial. Lancet.

[4] Llovet JM (2008). Sorafenib in advanced hepatocellular carcinoma. N. Engl. J. Med..

[5] Tsai WL (2020). Hepatic arterial infusion chemotherapy vs transcatheter arterial embolization for patients with huge unresectable hepatocellular carcinoma. Medicine (Baltimore).

[6] Guo JH (2020). Transarterial chemoembolization with hepatic arterial infusion chemotherapy plus S-1 for hepatocellular carcinoma. World J. Gastroenterol..

[7] Song MJ (2015). Hepatic artery infusion chemotherapy for advanced hepatocellular carcinoma. World J. Gastroenterol..

[8] Han S (2021). Treatment efficacy by hepatic arterial infusion chemotherapy vs. sorafenib after liver-directed concurrent chemoradiotherapy for advanced hepatocellular carcinoma. J. Cancer Res. Clin. Oncol..

[9] Ikuta S, Aihara T, Yamanaka N (2018). Efficacy of sequential sorafenib plus hepatic arterial infusion chemotherapy in patients with Barcelona Clinic Liver Cancer stage B and C hepatocellular carcinoma: A retrospective single-institution study. Contemp. Oncol. (Pozn).

[10] Arizumi T (2015). Validation of a modified substaging system (Kinki criteria) for patients with intermediate-stage hepatocellular carcinoma. Oncology.

[11] Zou JH, Zhang L, Ren ZG, Ye SL (2016). Efficacy and safety of cTACE versus DEB-TACE in patients with hepatocellular carcinoma: A meta-analysis. J. Dig. Dis..

[12] Bargellini I (2021). Duration of response after DEB-TACE compared to lipiodol-TACE in HCC-naïve patients: A propensity score matching analysis. Eur. Radiol..

[13] Golfieri R (2014). Randomised controlled trial of doxorubicin-eluting beads vs conventional chemoembolisation for hepatocellular carcinoma. Br. J. Cancer.

[14] Arai Y, Kido C, Ariyoshi Y (1993). Pharmacokinetics in arterial infusion chemotherapy. Gan Kagaku Ryoho Cancer Chemother..

[15] Collins JM, Dedrick RL (1982). Contribution of lungs to total body clearance: Linear and nonlinear effects. J. Pharm. Sci..

[16] Zimm S, Collins JM, O'Neill D, Chabner BA, Poplack DG (1983). Inhibition of first-pass metabolism in cancer chemotherapy: Interaction of 6-mercaptopurine and allopurinol. Clin. Pharmacol. Ther..

[17] Obi S, Sato S, Kawai T (2015). Current status of hepatic arterial infusion chemotherapy. Liver Cancer.

[18] Nishikawa H, Osaki Y, Kita R, Kimura T (2012). Hepatic arterial infusion chemotherapy for advanced hepatocellular carcinoma in Japan. Cancers (Basel).

[19] Guo W (2020). Efficacy and safety of hepatic arterial infusion chemotherapy combined with transarterial embolization for unresectable hepatocellular carcinoma: A propensity score-matching cohort study. JGH Open.

[20] Hsu SJ (2021). Hepatic arterial infusion chemotherapy with modified FOLFOX as an alternative treatment option in advanced hepatocellular carcinoma patients with failed or unsuitability for transarterial chemoembolization. Acad. Radiol..

[21] Kawaoka T (2018). Comparison of hepatic arterial infusion chemotherapy between 5-fluorouracil-based continuous infusion chemotherapy and low-dose cisplatin monotherapy for advanced hepatocellular carcinoma. Hepatol. Res. Off. J. Jpn. Soc. Hepatol..

[22] Moriya K (2018). Efficacy of bi-monthly hepatic arterial infusion chemotherapy for advanced hepatocellular carcinoma. J. Gastrointest. Oncol..

[23] Kondo Y, Fukuda R (2020). Cutting edge of hepatic artery infusion chemotherapy for hepatocellular carcinoma. Gan Kagaku Ryoho Cancer Chemother..

[24] Ni JY (2018). Transcatheter hepatic arterial infusion chemotherapy vs sorafenib in the treatment of patients with hepatocellular carcinoma of Barcelona Clinic Liver Cancer stage C: A meta-analysis of Asian population. OncoTargets Ther..

[25] Sung PS (2019). Reduction of intrahepatic tumour by hepatic arterial infusion chemotherapy prolongs survival in hepatocellular carcinoma. Anticancer Res..

[26] Iwamoto H (2021). Survival benefit of hepatic arterial infusion chemotherapy over sorafenib in the treatment of locally progressed hepatocellular carcinoma. Cancers (Basel).

[27] Nagamatsu H (2010). Intra-arterial therapy with cisplatin suspension in lipiodol and 5-fluorouracil for hepatocellular carcinoma with portal vein tumour thrombosis. Aliment. Pharmacol. Ther..

[28] Hatooka M (2018). Hepatic arterial infusion chemotherapy followed by sorafenib in patients with advanced hepatocellular carcinoma (HICS 55): An open label, non-comparative, phase II trial. BMC Cancer.

[29] Kudo M (2015). Subclassification of BCLC B stage hepatocellular carcinoma and treatment strategies: Proposal of modified Bolondi's subclassification (Kinki criteria). Dig. Dis..

[30] Li MF, Leung HW, Chan AL, Wang SY (2018). Network meta-analysis of treatment regimens for inoperable advanced hepatocellular carcinoma with portal vein invasion. Ther. Clin. Risk Manag..

[31] Zheng K (2022). Sorafenib plus hepatic arterial infusion chemotherapy versus sorafenib for hepatocellular carcinoma with major portal vein tumor thrombosis: A randomized trial. Radiology.

[32] He M (2019). Sorafenib plus hepatic arterial infusion of oxaliplatin, fluorouracil, and leucovorin vs sorafenib alone for hepatocellular carcinoma with portal vein invasion: A randomized clinical trial. JAMA Oncol..

[33] Ahn YE (2021). Comparison of sorafenib versus hepatic arterial infusion chemotherapy-based treatment for advanced hepatocellular carcinoma with portal vein tumor thrombosis. Gut Liver.

[34] Kodama K (2018). Comparison of outcome of hepatic arterial infusion chemotherapy combined with radiotherapy and sorafenib for advanced hepatocellular carcinoma patients with major portal vein tumor thrombosis. Oncology.

[35] Choi C, Yoo GS, Cho WK, Park HC (2019). Optimizing radiotherapy with immune checkpoint blockade in hepatocellular carcinoma. World J. Gastroenterol..

[36] Tang R (2020). Ferroptosis, necroptosis, and pyroptosis in anticancer immunity. J. Hematol. Oncol..

[37] Wang Y (2020). The safety and efficacy of lenvatinib combined with immune checkpoint inhibitors therapy for advanced hepatocellular carcinoma. Biomed. Pharmacother..

[38] Mizukoshi E (2016). Myeloid-derived suppressor cells correlate with patient outcomes in hepatic arterial infusion chemotherapy for hepatocellular carcinoma. Cancer Immunol. Immunother. CII.

[39] Saeki I (2018). Evaluation of the "assessment for continuous treatment with hepatic arterial infusion chemotherapy" scoring system in patients with advanced hepatocellular carcinoma. Hepatol. Res. Off. J. Jpn. Soc. Hepatol..

[40] Saeki I (2019). Effect of body composition on survival benefit of hepatic arterial infusion chemotherapy for advanced hepatocellular carcinoma: A comparison with sorafenib therapy. PLoS ONE.

[41] Takaya H (2020). Association between ADAMTS13 activity-VWF antigen imbalance and the therapeutic effect of HAIC in patients with hepatocellular carcinoma. World J. Gastroenterol..

[42] Terashima T (2020). IL-28B variant as a predictor in patients with advanced hepatocellular carcinoma treated with hepatic arterial infusion chemotherapy. J. Gastroenterol. Hepatol..

[43] Tsunematsu S (2017). Combination of neutrophil-to-lymphocyte ratio and early des-γ-carboxyprothrombin change ratio as a useful predictor of treatment response for hepatic arterial infusion chemotherapy against advanced hepatocellular carcinoma. Hepatol. Res. Off. J. Jpn. Soc. Hepatol..

[44] Wada F (2018). High expression of CD44v9 and xCT in chemoresistant hepatocellular carcinoma: Potential targets by sulfasalazine. Cancer Sci..

[45] Yamamoto S (2020). The early decline of α-fetoprotein and des-γ-carboxy prothrombin predicts the response of hepatic arterial infusion chemotherapy in hepatocellular carcinoma patients. Gastrointest. Tumors.

[46] Iwata T (2016). PD-L1(+)MDSCs are increased in HCC patients and induced by soluble factor in the tumor microenvironment. Sci. Rep..

